# Phase Retardation Analysis in a Rotated Plane-Parallel Plate for Phase-Shifting Digital Holography

**DOI:** 10.3390/jimaging8040087

**Published:** 2022-03-24

**Authors:** Igor Shevkunov, Nikolay V. Petrov

**Affiliations:** 1 Faculty of Information Technology and Communication Sciences, Tampere University, 33100 Tampere, Finland; 2 Digital and Display Laboratory, ITMO University, 197101 St. Petersburg, Russia; n.petrov@niuitmo.ru

**Keywords:** digital holography, phase-shifting digital holography, phase measurement, phase imaging, plane parallel plate

## Abstract

In this paper, we detail a phase-shift implementation in a rotated plane-parallel plate (PPP). Considering the phase-shifting digital holography application, we provide a more precise phase-shift estimation based on PPP thickness, rotation, and mutual inclination of reference and object wavefronts. We show that phase retardation uncertainty implemented by the rotated PPP in a simple configuration is less than the uncertainty of a traditionally used piezoelectric translator. Physical experiments on a phase test target verify the high quality of phase reconstruction.

## 1. Introduction

Phase-shifting interferometry is a technique of complex amplitude measurement [1]. It is based on the registration and processing of several holograms exposed to phase-shifted reference wavefronts. Its original analog version is very laborious because of complex processing. However, with the development of matrix photo-detectors [2], digital phase-shifting interferometry [3] and phase-shifting digital holography (PSDH) [4] are immensely improved due to the digital implementation [5,6], since now all complex processing (such as registration, developing, and reconstruction) is performed on computers. Due to its straightforward computational realization, high resolution, and precise elimination of spurious zero and minus first diffraction orders in the in-line configuration [7], PSDH has become a popular method for complex amplitude reconstruction.

Since Yamaguchi and Zhang [8] published the first work on phase-shifting digital holography for four phase shifts of π/2, many different implementations of phase shifts and their subsequent processing [9,10] have been proposed. In that way, reductions in the number of the steps to three [11,12] and two [13,14] have been proposed. Furthermore, regarding the phase step values, automatic estimation for unknown values was proposed in works [15,16,17].

Regarding the phase shift implementation as it was realized in the pioneering paper, the most popular one is a piezoelectric translator (PZT). It moves a reference arm mirror with nano-scale precision and, because of a change in the actual length of a light path, the desired phase delay is achieved. Likewise, for this purpose but without mechanical movements, quarter-wave plates [18,19], liquid phase-retarders [13,20,21,22], and spatial light modulators (SLM) [23,24] have been used. While SLM is an expensive solution with a pixelated structure that might decrease imaging resolution, liquid phase-retarders are demanding in terms of precise optical adjustment, since light changes its polarization during retardation.

In papers [25,26,27,28,29,30], phase-shifting with plane-parallel plate (PPP) rotation was described, where a phase-shift was obtained due to path lengthening in the PPP because of its rotation. However, the phase retardation in the rotated PPP was only mentioned without a complete description of the process. Moreover, in [28] phase retardation analysis was performed with trigonometric simplification, which does not correspond to the correct value of the phase delay, and in [29] the equation was published with a misprint.

In PSDH, as in all metrological techniques, the reconstruction quality strongly depends on the quality of an optical system and noise. Noise especially influences the phase measurement in PSDH because of several observations with different phase shifts, resulting in a superposition of noise from each observation. Different factors of the registration system introduce noise in PSDH: an inaccuracy in the phase shift determination, vibration distortion, and sensor noise [20,31,32]. It was found in [32] that the largest distortion of the reconstructed wavefront is introduced by errors associated with the inaccuracy of the phase shift determining, i.e., phase-shift implementation. In papers [33,34,35,36,37] the errors in determining the value of the phase-shift were considered with solutions to reduce these errors. However, these solutions were concentrated on an inhibition of the consequences of the incorrect phase-shift implementations not considering the cause. In the current paper, we aim to investigate the cause of the phase-shift inaccuracy in the rotated PPP and show that this inaccuracy is less than in PZT, which is traditional for PSDH. We thereby provide an effective equivalent to PZT for phase-shift implementation.

## 2. Phase Retardation in a Rotated Plane-Parallel Plate

An optical plane-parallel plate is a transparent body bounded by two mutually parallel polished surfaces. When placed in the optical path it preserves the light ray direction because of refraction on both surfaces [30]. However, in the case where the PPP is inclined to the optical path (rotated on its axis), the effects of optical path lengthening and lateral displacement of the light ray appear due to refraction on the first surface. Considering both effects, we provide precise phase-shift estimation caused by the rotated PPP.

**Figure 1:**
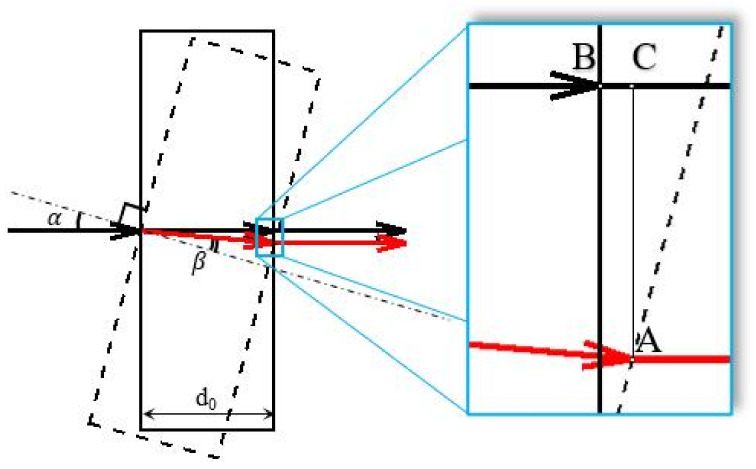
Path difference in plane-parallel plate. α is the PPP rotational angle, the black arrow is the light path with α=0, the red arrow is a light path with α≠0, $d_{0}$ is the PPP thickness, and β is the angle of refraction. Zoomed inset shows the detailed location of the shifted light path because of PPP rotation.

Figure 1 shows light ray propagation through the PPP. Black arrows illustrate the light path corresponding to the PPP’s initial position (solid black rectangle) with angle α=0 and red arrows corresponding to the light path in the rotated PPP (dashed rectangle) when α≠0, $d_{0}$ is the PPP thickness and β is the refraction angle. The optical path of a light ray in the PPP’s initial position is $n\cdot d_{0}$, where *n* is the refractive index of the glass.

To estimate the phase-shift obtained by a wavefront going through the rotated PPP, one needs to subtract the wavefront phases after and before the rotation. For detailed investigation of the accumulated phase shift, we point out the points A,B, and *C* in the zoomed inset in Figure 1: if α=0 the light path goes out from the PPP in the point *B*, and after PPP rotation, it comes out from the point *A*. As can be seen from the figure, point *A* is located further along the optical path than point *B*, therefore for the correct phase shift estimation, the phase difference must be calculated between points *A* and *C*. Thus, length BC must be considered. In addition, taking into account the PSDH application where reference and object wavefronts create a hologram, mutual spatial displacement (length AC) of these wavefronts will result in an additional phase shift if the reference and object wavefronts are inclined to each other. When the angle θ between the reference and object wavefronts equals zero, there is no lateral phase-shifting effect, but in physical experimental conditions, it is hard to attain a zero value for θ.

Therefore, to estimate the resulting phase-shift caused by the rotation of the PPP, we have to find the phase difference between wavefronts in points *A* (after rotation) and *C* (before rotation) following equation

(1) $$\begin{array}{cc} \Delta \varphi & =\varphi_{A}-\varphi_{C}+\varphi_{\theta };\\ \varphi_{A}=\frac{2\pi dn}{\lambda };\;\varphi_{C} & =\frac{2\pi }{\lambda }\left(d_{0}n+BC\right);\;\varphi_{\theta }=\frac{2\pi }{\lambda }AC\cdot \tan\theta , \end{array}$$

where Δφ is the desired phase delay, $\varphi_{A}$ is the wavefront phase in point *A*, $\varphi_{C}$ is the wavefront phase in point *C*, $\varphi_{\theta }$ is the phase shift because of inclination between the object and reference wavefronts, λ is the wavelength, *n* is the PPP refractive index, $d_{0}$ is the plate thickness, dn is the light path length in the PPP after rotation, and θ is the angle between the reference and object wavefronts. Considering all described phases and the law of refraction, we present the resulting equation for the phase shift estimation in the rotated PPP:

(2) $$\Delta \varphi =\frac{2\pi d_{0}}{\lambda }\left[\frac{n}{\cos\beta }-\left(n+\frac{\cos(\alpha -\beta )}{\cos\beta }-1\right)+\tan\theta \cdot \frac{\sin(\alpha -\beta )}{\cos\beta }\right].$$

## 3. Phase Retardation Analysis

**Figure 2:**
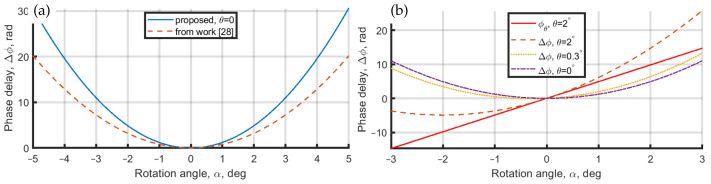
Phase delay in rotated PPP. (**a**) Phase delay with no inclination among reference and object wavefronts. The solid blue line is for the proposed phase delay estimation, dashed orange curve is for phase delay from the paper [28]. (**b**) Phase delay dependence on θ inclination angle between reference and object wavefronts. The solid red line is phase shift, $\varphi_{\theta }$ because of inclination on $\theta =2^{\circ }$, a dotted orange curve is phase delay with θ=0, a dash-dot purple curve is phase delay with $\theta =0.3^{\circ }$, and dashed orange curve is phase delay with $\theta =2^{\circ }$.

To illustrate the difference of the proposed equation with the previous phase-shift estimation [28] in the rotated PPP, we have plotted in Figure 2a these phase-shifts depending on rotation angle, α. For clarity, we consider the ideally in-line geometry, which means that the angle between the reference and object wavefronts equals zero (θ=0), with the PPP thickness $d_{0}=2$ mm, n=1.5163, and λ=532 nm. In Figure 2a, the solid blue curve is for the proposed phase shift estimation and the dashed orange curve is for the estimation from the paper [28]. In the work [28], the authors provide the equation $\frac{2\pi d_{0}}{\lambda n}\left(n\cos\beta -\cos\alpha \right)$, which is based on trigonometrical simplification and estimates the phase-shift, which is not correct and is smaller than the direct estimation.

Considering the case when the reference and object wavefronts are inclined to each other (θ≠0), the $\varphi_{\theta }$ in Equation (1) creates an almost linear shift of the total phase delay, Δφ; see Figure 2b, where we have plotted two curves of phase shift in PPP with θ=0 in purple, θ=0.3 in yellow, and with θ=2 deg in orange. A solid red line corresponds to the $\varphi_{\theta }$ phase with θ=2deg. The $\varphi_{\theta }$ curve is linear; therefore, it only shifts the resulting phase delay Δφ without changing its shape. It is seen that θ=2deg shifts the minimum of Δφ from α=0deg to α=−2deg and even small values of θ (e.g., 0.3deg) will influence the resulting phase-shift and must be considered for precise estimation of the phase-shift.

## 4. Uncertainty Estimation

**Figure 3:**
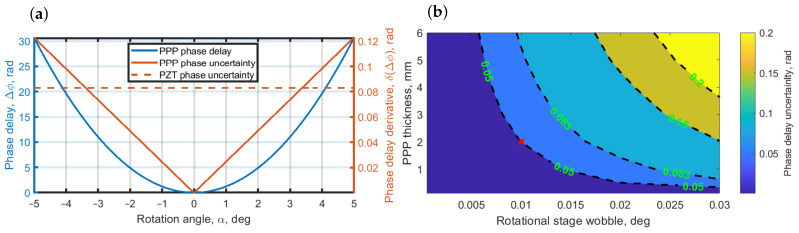
Phase-shift precision estimation in PPP and PZT. (**a**) Left *y*-axis is for the phase delay; right *y*-axis is for the phase delay uncertainty. The solid blue curve is the phase delay in the rotated PPP, the solid orange curve is the uncertainty of the phase delay in the PPP, and the dashed orange line is the uncertainty of PZT. (**b**) Rotated PPP phase delay uncertainty map for delay value of (3π/2+1) rad depending on PPP thickness and rotational stage wobble. Red dot corresponds to the considered MTS wobble of 0.01 deg and PPP thickness of 2 mm.

Next, to estimate the stability of the phase delay in PPP, we show in Figure 3 the PPP’s phase delay uncertainty. For the sake of clarity, we have taken θ=0. The blue curve in Figure 3a illustrates the phase delay dependence on the PPP rotation angle, α; the solid orange curve is the uncertainty estimation that was calculated as a derivative of the phase delay. The dashed orange curve is the phase uncertainty of the traditional phase-shifting implementation—a piezoelectric translator (PZT). The PPP and PZT uncertainties were calculated based on the parameters of the instruments available in our laboratory. For the rotated PPP, this was the rotatable motorized translation stage (MTS) “Standa mr151-30” (https://www.standa.lt/products/catalog/motorised_positioners?item=9&prod=motorized_rotation_stages&print=1, access date 1 March 2022), in which the smallest rotation step is 0.01 degree and the repeatability equals the step value. For PZT, we estimated the uncertainty based on PZT “Physik Instrumente P-603.1s2” (https://www.physikinstrumente.store/eu/p-603.1s2/, access date 1 March 2022), with a wobble of 7 nm, which resulted in a phase uncertainty of 0.083 rad for the wavelength of 532 nm. Due to the nonlinearity of Equation (2), the PPP uncertainty increases with the rotational angle. In Figure 3a it can be seen that for rotation angles in the range [−3.1:3.1] deg, PPP uncertainty is less than for PZT. Therefore, PPP is more stable in that range. For angles of rotation larger than 3.1deg PPP will perform worse; however, PPP rotation angle in that range corresponds to a phase delay of about [0:13] rad, which is more than enough for PSDH reconstruction where only a 3π/2 maximum phase-shift is needed.

Mainly, the rotated PPP uncertainty is a function of two parameters: the wobble of a rotational stage and PPP thickness. To estimate the uncertainty for the realization of the required 3π/2 phase delay, we show in Figure 3b an uncertainty contour map of the rotated PPP for the phase delay value of (3π/2+1) depending on both PPP thickness and MTS wobble. The contours of 0.083 rad and of 0.05 rad correspond to PZT of 7 nm and 4.2 nm wobble, respectively. As can be seen from the map in Figure 3b, a thinner PPP provides smaller errors even with relatively high MTS wobble. The red dot in Figure 3b is for the considered MTS wobble of 0.01 deg and PPP thickness of 2 mm, which corresponds to PZT with a 4.2 nm wobble. Contrary to PZT, MTS has less precision, and to introduce a sufficient phase delay the PPP has to be rotated on a relatively large angle, thus small instabilities in MTS do not impact the phase retardation. Therefore, the proposed implementation of phase retardation surpasses PZT in terms of stability.

## 5. Experimental Verification

The basic PSDH method reconstructs the object phase from four holograms recorded with shifted phases of the reference beam on 0, π/2, π, and 3π/2 [8]. One hundred holograms of each phase step are averaged in order to smooth the noise of the sensor and vibrations. To calculate the phase one must follow the equation

(3) $$\varphi \left(x,y\right)=\arctan\left[\frac{I_{3\pi /2}-I_{\pi /2}}{I_{0}-I_{\pi }}\right],$$

where φ(x,y) is the object phase and $I_{p}$ is the hologram with a phase-shifted reference beam. Such an approach is straightforward and cheap in terms of computational cost.

**Figure 4:**
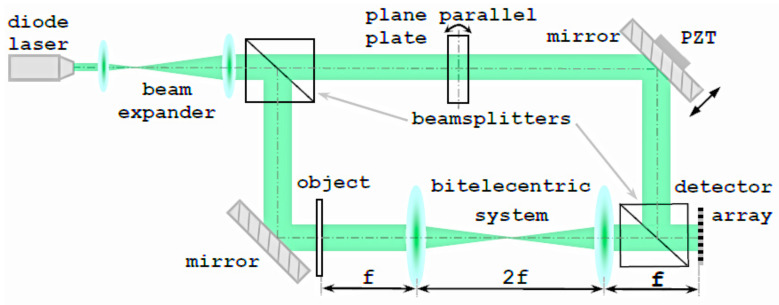
Experimental setup.

To compare PSDH reconstructions realized by the proposed rotated PPP and by traditional PZT implementations, we developed the experimental setup with PPP and PZT phase shifts implemented simultaneously. It is presented in Figure 4 and based on a Mach–Zehnder interferometer in the in-line configuration. For phase-shift initialization, a rotation MTS Standa mr151-30 for PPP and PZT “Physik Instrumente P-603.1s2” were utilized. The radiation source was a single-mode laser Lasos CKL 2400, λ=532 nm; the detector array was the CMOS matrix VEI-830 with resolution 2048×1536 and pixel size Δx=1.4μm; PPP thickness $d_{0}$ equals 2 mm.

**Figure 5:**
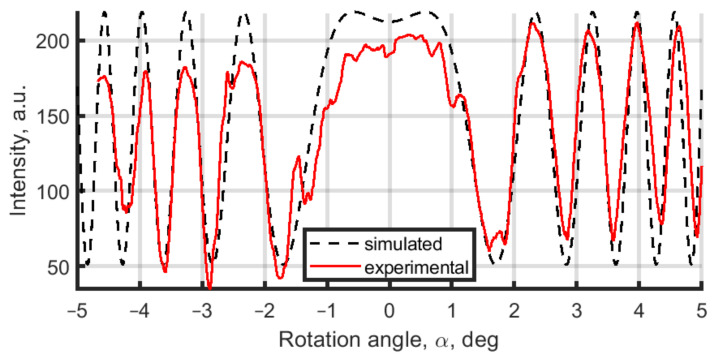
Hologram’s single pixel intensity dependence from rotational angle of the plane parallel plate.

Following Equation (2), to have the desired phase shifts of 0, π/2, π, and 3π/2, the PPP should be rotated on angles α=0,1.132,1.601,1.961deg, correspondingly. However, considering that even a small inclination between reference and object wavefronts might shift the expected phase delay, we performed an experimental calibration of the PPP phase delay system. The calibration was realized by scanning the PPP rotation angle in the range [−5:5] deg with a consecutive recording of the single-pixel hologram intensity [34]. Thus we present in Figure 5 the solid red curve of that single pixel intensity depending on the PPP rotation angle. The phase-shifts can be estimated by analyzing this curve: the maximums correspond to 2π·m phase-shifts, minimums correspond to π·m, and halves of the difference of maximum and minimum correspond to π/2·(2m+1) with an integer *m*. Phase-shift simulation in Figure 5 (dash black curve) is calculated by Equation (2), and it is in good agreement with the experimental data.

**Figure 6:**
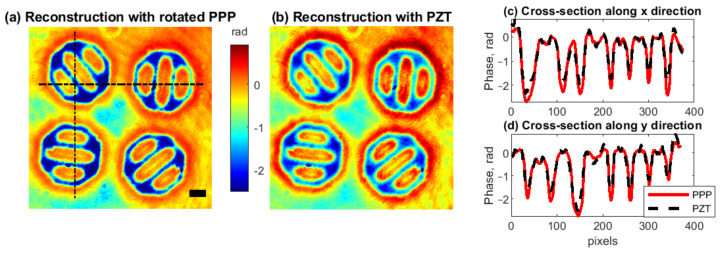
PSDH reconstructed phase with implementation by rotated PPP (**a**) and by PZT (**b**). Longitudinal cross-sections are in subplots (**c**,**d**) for *x* and *y* directions, correspondingly. Scale bar is 50 μm.

Figure 6 shows PSDH reconstruction of a phase object by PPP and PZT implementations. For comparison of these reconstructions, we built longitudinal cross-sections in Figure 6c,d. Visually, reconstructions are close to each other with the same spatial resolution. However, in cross-sections, phase deflections of about 0.3 rad are observed. From the theoretical uncertainty estimation in Section 4, we expect that the rotated PPP implementation should perform better. However, we cannot conclude unambiguously that this deflection is the error of PZT implementation since it might be addressed to the influence of noise, e.g., vibrations. Both techniques provide quality imaging with about 10% difference in phase from each other. At least we may conclude that PSDH phase reconstruction with rotated PPP implementation is a cost-effective equivalent for PZT.

## 6. Conclusions

In this paper, the model of phase-shift estimation in the PSDH with a rotated PPP has been further developed. We have detailed the mathematical equation for phase-shift estimation in the PPP, taking reference and object wavefront mutual inclination into account. Based on the equation, we have estimated the uncertainty of the phase-shift in the rotated PPP and found that PPP implementation is more stable than PZT in a wide range of parameters. It was shown that phase delay will be smaller with a thinner PPP. The thinner PPP thus guarantees higher precision in phase-shift estimation. Moreover, thin optical elements are on-demand in tasks, where embedding of additional elements to the optical scheme is undesirable, to eliminate dispersion broadening. Such restrictions take place in femtosecond holography [38,39,40] where the proposed implementation might now be applied. Additionally, PPP rotation might be realized in multicolor holography with color filters on a registration matrix [41,42,43]. The simplicity and cost-effectiveness of the considered phase-shifter allows widespread use of PSDH, overcoming the PZT technical barrier without loss in the reconstruction quality.

## Data Availability

Data available through request.

## Abbreviations

The following abbreviations are used in this manuscript:

PSDH	Phase-shifting digital holography
PPP	Plane-parallel plate
PZT	Piezoelectric translator
MTS	Motorized translation stage
SLM	Spatial light modulator
